# Exploring the implementation and underlying mechanisms of centralized referral systems to access specialized health services in Quebec

**DOI:** 10.1186/s12913-021-07286-3

**Published:** 2021-12-16

**Authors:** Jessica Spagnolo, Mylaine Breton, Martin Sasseville, Carine Sauvé, Jean-François Clément, Richard Fleet, Marie-Claude Tremblay, Cloé Rodrigue, Camille Lebel, Marie Beauséjour

**Affiliations:** 1 Department of Community Health Sciences, Faculty of Medicine and Health Sciences, Université de Sherbrooke, 150, Place Charles-Le Moyne, C. P. 200, Longueuil, QC J4K 0A8 Canada; 2 Centre de recherche Charles-LeMoyne, Université de Sherbrooke – Campus Longueuil, 150, place Charles-Le Moyne, C. P. 200, Longueuil, QC J4K 0A8 Canada; 3 Centre intégré de santé et de services sociaux (CISSS) de la Montérégie-Centre, 3141 Boulevard Taschereau Bureau 220, Greenfield Park, QC J4V 2H2 Canada; 4 Department of Family Medicine and Emergency Medicine, Faculty of Medicine and Health Sciences, Université de Sherbrooke, 2500 Boulevard de l’Université, Sherbrooke, QC J1K 2R1 Canada; 5 Department of Family and Emergency Medicine, Faculty of Medicine, Université Laval, Pavillon Ferdinand-Vandry, 1050, Avenue de la Médecine, Québec, QC G1V 0A6 Canada; 6 Centre de recherche en santé durable, Centre intégré universitaire de santé et services sociaux (CIUSSS) de la Capitale-Nationale, Pavillon Landry-Poulin, 2525 chemin de la Canardière, Québec, QC G1J 0A4 Canada; 7 Department of Surgery, Faculty of Medicine, Université de Montréal, C.P, 6128, succursale Centre-ville, Montréal, QC H3C 3J7 Canada

**Keywords:** Centralized referral mechanisms, Access to care, Specialized health services, Logic models, Health planning, Single-entry model, Quebec

## Abstract

**Background:**

In 2016, Quebec, a Canadian province, implemented a program to improve access to specialized health services (*Accès priorisé aux services spécialisés* (APSS)), which includes single regional access points for processing requests to such services via primary care (*Centre de répartition des demandes de services* (CRDS)). Family physicians fill out and submit requests for initial consultations with specialists using a standardized form with predefined prioritization levels according to listed reasons for consultations, which is then sent to the centralized referral system (the CRDS) where consultations with specialists are assigned. We 1) described the APSS-CRDS program in three Quebec regions using logic models; 2) compared similarities and differences in the components and processes of the APSS-CRDS models; and 3) explored contextual factors influencing the models’ similarities and differences.

**Methods:**

We relied on a qualitative study to develop logic models of the implemented APSS-CRDS program in three regions. Semi-structured interviews with health administrators (*n* = 9) were conducted. The interviews were analysed using a framework analysis approach according to the APSS-CRDS’s components included in the initially designed program, Mitchell and Lewis (2003)’s logic model framework, and Chaudoir and colleagues (2013)’s framework on contextual factors’ influence on an innovation’s implementation.

**Results:**

Findings show the APSS-CRDS program’s regional variability in the implementation of its components, including its structure (centralized/decentralized), human resources involved in implementation and operation, processes to obtain specialists’ availability and assess/relay requests, as well as monitoring methods. Variability may be explained by contextual factors’ influence, like ministerial and medical associations’ involvement, collaborations, the context’s implementation readiness, physician practice characteristics, and the program’s adaptability.

**Interpretation:**

Findings are useful to inform decision-makers on the design of programs like the APSS-CRDS, which aim to improve access to specialists, the essential components for the design of these types of interventions, and how contextual factors may influence program implementation. Variability in program design is important to consider as it may influence anticipated effects, a next step for the research team. Results may also inform stakeholders should they wish to implement similar programs to increase access to specialized health services via primary care.

**Supplementary Information:**

The online version contains supplementary material available at 10.1186/s12913-021-07286-3.

## Introduction

Accessing specialized health services is a challenge in Canada; mean wait times increased from 3.7 weeks in 1993 to 8.9 weeks in 2018 [1]. Specialized health care is defined in Canada as services provided by a specialist consultant, a physician who has completed full medical training in a specialized area of medicine and granted licence by the provincial or territorial Medical Regulatory Authorities, in hospital-based or community settings (including specialist visits, non-emergency surgery and diagnostic tests) [2]. According to the 2016 Commonwealth Fund survey conducted in 11 countries, Canadians reported longer wait times to access specialized health services than other comparable countries [3], and reports suggest that these are worsening in Canada [4]. In Quebec, a Canadian province, wait times to access specialized health services have also increased. Studies show that in 1993, the mean average wait time to see a specialist was 2.9 weeks, whereas in 2018, wait times increased to 6.7 weeks [1].

Quebec, like in other Canadian provinces and several countries worldwide [5], majorly relies on family physicians (FPs) to refer patients to specialized health services [6]. After completing a 4- or 5-year undergraduate doctorate in medicine, physicians in training aiming to practice family medicine complete a 2-year residence program accredited by the College of Family Physician in Canada [7]. Referrals from FPs to specialists are a necessary step for patients to access specialized health resources in countries with universal healthcare coverage. However, barriers to accessing timely specialized health services in a FP gatekeeper health system context can include the many steps involved for patients in accessing care and lack of specialists’ availability, which can increase service wait time and contribute to patient frustration [8, 9].

The Quebec government implemented a program to facilitate patient access to specialized health services via FP referrals in primary care settings (*Accès prioritaire aux soins spécialisés* (APSS) (English: Priority Access to Specialized Health Services Program)) [10]. The program includes a centralized referral system (*Centres de répartition des demandes de services* (CRDS)), single regional access points to process requests for first time specialized health service consultations, as well as specialists’ registration [11]. The APSS-CRDS was implemented in the context of Bill 20 [12], enforcing the program by law. It aimed to promote access to family medicine and specialized health services by “optimiz[ing] the utilization of the medical and financial resources of the health system [and] introduc[ing] certain obligations applicable to the practice of physicians who participate in the Quebec Health Insurance Plan” [13]. The *Fédération des médecins spécialistes du Québec* (FMSQ), the health specialists’ union, and the Quebec Ministry of Health and Social Services devised a tentative agreement to improve access to specialized medical services, which deferred the application of Bill 20 and allowed for its subsequent repeal to the extent that access targets were met [14]. They also agreed on a timeline for achieving these targets.

The APSS-CRDS was implemented in each of Quebec’s 18 administrative regions over three phases (October 2016 to March 2019), starting with selected specialties such as orthopedics, cardiology, neurology, and urology, to name a few [11]. It now includes 26 specialties. FPs fill out and submit requests for initial consultations with specialists using a standardized form with predefined prioritization levels according to listed reasons for consultations [15], which is then sent to the CRDS, where consultations with specialists are assigned.

The APSS-CRDS was designed as a centralized waiting list. Previous work by our team on centralized access to FPs suggests that the overall operation of centralized waiting lists may be conceptualized around three mechanisms: consolidation of patient demand through a central intake; triage and prioritization of the demand; and patient assignment to the most appropriate service provider among a pool of providers [16, 17]. The literature [18] recognizes different referral intervention typologies that can be used to describe the main features of the APSS-CRDS program. First, some interventions target education and feedback, as referring guidelines used as a stand-alone tool were shown to have low efficacy for patient referrals. However, providing physicians with reasons for rejection of the referral may improve referral decision processes.

The APSS-CRDS incorporates these principles by providing FPs with a list of pre-determined reasons for consultation (e.g., for orthopaedics: recurrent shoulder dislocation with physiotherapy initiated; for cardiology: asymptomatic bradycardia with heart rate < 40 bpm or documented daytime > 3 s pauses), pre-determined priority levels according to reasons for consultation (e.g., recurrent shoulder dislocation should be seen within 3 months; asymptomatic bradycardia within 10 days), as well as clinical alerts in the forms prompting referral to the emergency department (e.g., cauda equina syndrome; suspected acute coronary syndrome). In addition, feedback and support from medical advisors are provided to the FPs for “non-standard” patients’ registration and prioritisation (i.e., patients intended to be referred, but who do not meet the listed reasons for consultation), and administrative conformity and clinical validation processes conducted by nurses and/or administrative agents [15]. Internationally, other models incorporate similar features in centralized referral mechanisms to refer patients from primary to specialized health care [19–22].

Second, Blank et al. [18] also highlight process change interventions. The APSS-CRDS adopt these intervention features by relying on standardized forms with pre-requisites for referrals that FPs can complete (e.g., an ultrasound or MRI report and treatment failure are required before referring a patient for epicondylitis; a conforming EMG report is required before referring a patient for carpal tunnel) and a single-entry model (e.g., referrals by FPs working in primary care settings). Other examples use such features [23–25].

Third, the APSS-CRDS includes features belonging to “system change” referral interventions from Blank and colleagues' [18] typology, and these work at macro/meso levels. Examples of these types of demand management interventions include programs that aim to manage waiting lists like surgical care networks (implemented in the Canadian provinces of Saskatchewan, Alberta, British Columbia) [26–28]; provincial coordination and prioritization registries [29]; and interventions that aim to find the most appropriate specialized health resources at the regional level to coordinate care with patients [30]. The APSS-CRDS includes such intervention features by relying on homogenous waiting groups (i.e., clinical conditions linked to maximum waiting times according to a pre-determined ranking system [19]), regional referral management centres, and the coordination of the regional service offer. In addition, the APSS-CRDS includes from these types of models features that allow for the allocation of patients to the first qualified and available specialist in the region, the centralized management of specialist appointments and direct booking with specialists, as well as target monitoring (e.g., number of appointments provided within the target delays are monitored and reported to the Quebec Ministry of Health and Social Services) [14, 15]. In Canada, wait times are subject to a pan-Canadian follow-up for certain medical conditions: hip fracture repair, cardiac surgery, cataract surgery, knee or hip replacement, and access to radiotherapy [31]. Such monitoring strategies are also implemented at the regional level in the APSS-CRDS program, and this for homogeneous waiting groups in each specialty [14, 15].

Recent reports highlight challenges in achieving APSS-CRDS target delays for clinical priorities and variability in the program’s implementation and use across regions [32, 33], which to our knowledge are currently not formally evaluated. Emerging literature on centralized wait lists considers how these models are implemented and how they work across different contexts, as well as how these varying contexts can influence the reaching of anticipated effects [17, 25, 34–36].

This paper’s overarching objective is to build on these efforts to better understand the APSS-CRDS models and their variability, a first step in the research’s team ability to better understand how variations in the implementation, operation, and use of the APSS-CRDS can influence target outcomes. Specifically, we aimed to 1) describe the APSS-CRDS program in three Quebec regions using logic models; 2) compare similarities and differences in the components and processes of the APSS-CRDS models; and 3) explore contextual factors influencing the models’ similarities and differences.

## Methods

### Study design

This paper is part of a larger research project aiming to better understand the implemented APSS-CRDS models and their operation from the perspective of health managers (*n* = 9) and their use by FPs (*n* = 10) in three Quebec regions [37]. For this article, we relied on a qualitative descriptive design [38, 39], which helped in better understanding health manager perspectives on the APSS-CRDS to develop logic models of the program in three Quebec regions.

### Study settings and participants

We conducted this study in three Quebec regions, which were purposefully selected as they display variation in characteristics. Their description is included in Table 1 [40].

**Table 1:** Region size and health care organization of the Quebec study regions

	Region A	Region B	Region C
**Region size**	-Population: 2,069,849 inhabitants	-Population: 1,603,232 inhabitants	-Population: 525,684 inhabitants
	-Density: 4155 inhabitants/square km	-Density: 144 inhabitants/square km	-Density: 12 inhabitants/square km
**Health care organization**	-5 CIUSSS^a^ regrouping 15	-3 CISSS^b^	-1 CIUSSS^a^
	hospital centres and 5 non-merged institutions	regrouping 9 hospital centres	regrouping 6 hospital centres
	-3,29 physicians/1000 inhabitants	-1,71 physicians/1000 inhabitants	-2,07 physicians/1000 inhabitants

^a^*Centres intégrés universitaires de santé et de services sociaux* (English: University Health and Social Services Centres) regroup hospital centres, clinics, group homes, child protection centres, and rehabilitation centres. It is in a health region where a university offers a full undergraduate medical program and/or operates a center designated as a university institute in the health and/or social fields

^b^*Centres intégrés de santé et de services sociaux* (English: Integrated Health and Social Services Centres) regroup hospital centers, clinics, group homes, child protection centres, and rehabilitation centres

To recruit health administrators involved in implementing the APSS-CRDS program, we contacted the APSS-CRDS chief of service and inquired about potential interviewees. The chief of service then invited health administrators. In total, nine agreed to participate in semi-structured interviews: 2 from Region A; 3 from Region B; and 4 from Region C.

### Data collection

We chose Mitchell & Lewis (2003)’s framework to guide the development of the logic models [41]. Specifically, it was chosen as a methodological tool to help us collect, analyze, and organize data that describes the mechanisms (strategies-processes-outcomes) of the APSS-CRDS’s operation. Hence, with the use of the logic model tool, we were able to translate conceptual knowledge about participant experiences with the APSS-CRDS to a visual structure that synthesized the program per study region. Details between regions could then be compared across regions to glean similarities and differences that may be influenced by comparable or differing contexts. Cross-comparison of findings between developed logic models will thus help to explain variability, and to better understand links between context and program design.

Logic models as a methodological tool were used previously in a comparable manner to study the functioning of prioritization and allocation mechanisms for centralized waiting lists in Canada [42]. The logic model [41] components are included in Table 2.

**Table 2:** Mitchell & Lewis (2003)’s [41] logic model components

Components	Description
Action areas	“The broad focus” of the intervention
Outcome areas	Changes the intervention is “trying to bring about for individuals, communities, and/or service systems”
Input and Strategies	Resources and activities needed for the intervention
Processes and Structures	“Service and service system characteristics that are considered necessary to bring about lasting impacts on target individuals, communities, and/or service systems”
Intended Impacts	“Changes anticipated for individuals, communities, and/or service systems” because of the intervention and measured by for example performance indicators

**Fig. 1:**
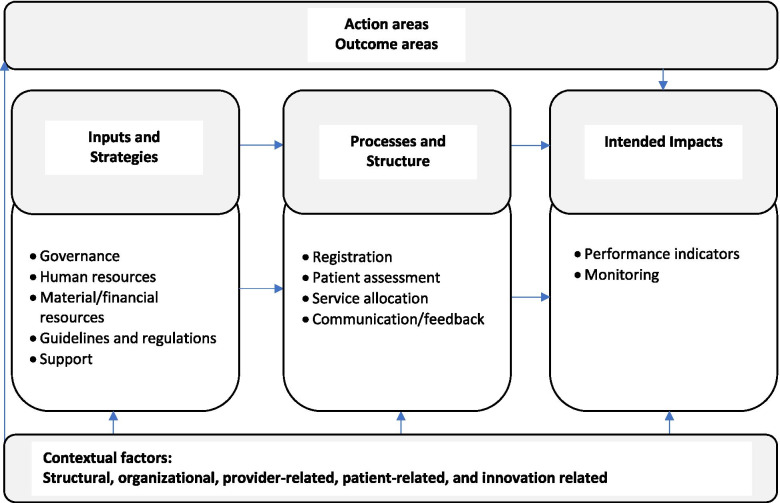
Multi-factor framework

We complemented the logic model framework [41] with Chaudoir and colleagues (2013)’s framework, and this to guide the identification/categorization of contextual factors [43] related to the APSS-CRDS program’s variability and to increase our capacity to conceptualize the constructs hypothesized to influence implementation. These contextual factors are treated in a more global fashion in Mitchell and Lewis (2003)’s framework [41]. Chaudoir and colleague [43]’s framework includes five categories: 1) structural factors like Quebec’s socio-political context (broader social, historical, and cultural factors); 2) organizational factors like regional and/or local leadership, and collaborations; 3) provider factors like characteristics that might influence physicians’ use of the APSS-CRDS; 4) innovation factors like context readiness and program adaptability; and 5) patient factors such as patient characteristics and benefits of the program’s use. Regional logic model content was additionally informed by APSS-CRDS ministerial guidelines for the management of consultation requests (components and processes), included in the Quebec Ministry of Health and Social Services CRDS Management Guide [15]. These include components adapted to consider the general components/functions proposed by the Quebec Ministry of Health and Social Services [15] and the main mechanisms of centralized waiting lists [16, 28], which comprise the themes listed in ‘processes and structures’ (Fig. 1). Hence, our multi-factor framework (Fig. 1) contains a mix of two frameworks (Mitchell and Lewis [41] and Chaudoir and colleagues [43]), as well as the APSS-CRDS general components proposed by the Quebec Ministry.

To inform the logic models, we conducted nine interviews with key stakeholders between August 2019 and January 2021. Questions were structured according to Mitchell & Lewis’ logic model framework [41] and Chaudoir and colleagues [43]’ framework, and included components of the APSS-CRDS program listed in the Quebec Ministry of Health and Social Services CRDS Management Guide [15]. Three interviews were conducted individually and three were conducted with two participants working in the same service. Interviews lasted between 60 and 100 min, and were conducted in French by the third and last authors.

The interview guide with open-ended questions was developed in French by the first, third and last authors (Supplementary File 1, translated from French by the authors). Prior to its use, a first version of the interview guide was piloted with two chiefs of service in hospital specialized care to validate the relevance of the questions. The content and wording of the interview guide was then revised, simplified, and shortened, and satisfactorily retested with an APSS-CRDS manager and a regional access to care manager.

### Data analysis and scientific rigor

Framework analysis [44, 45] on interview transcriptions was conducted using deductive and inductive approaches [46]. We selected this approach as the goal was to reduce our data from each region into a hierarchy of themes and sub-themes included in pre-determined frameworks, all the while retaining links with the original data. Hence, the logic model is the summarized mapping of the data and interpretation of the findings includes an inventory of themes within a region and a theme-based comparison between regions. To accomplish this, first, the interview guide helped to identify preliminary themes, sub-themes, and codes [47] that matched the APSS-CRDS program components and processes, Mitchell and Lewis (2003)’s logic model components [41], and Chaudoir and colleagues (2013)’s contextual factor categories [43] and allowed for the development of a preliminary code book [48] by the first and third authors. Of note, this codebook was developed in English. Second, one transcript was coded as a group (with the first, third, and last authors) using the preliminary code book. New codes were identified and added to the code book. Disagreements were managed by drafting definitions of sub-themes and codes and then discussing the verbatim accordingly, until agreement was met. Third, with the new code book, the first author proceeded to code the remaining transcripts and the third author coded one. Last, the first and last authors then worked together to aggregate certain matching codes and examples to populate the sub-themes of the logic model framework. The coding process was verified by the first and last authors at multiple steps, to ensure validity of the process and content (multiple examiners, [49]). The few disagreements had in this verification process were resolved by clarifying definitions of codes and verbatim, resulting in reassigning verbatim to other more appropriate codes and/or refining codes.

**Fig. 2:**
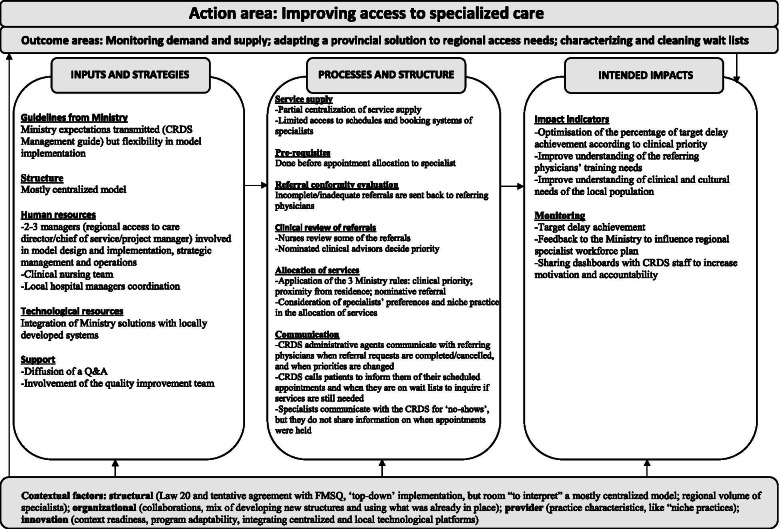
Region A (mostly centralized model)

**Fig. 3:**
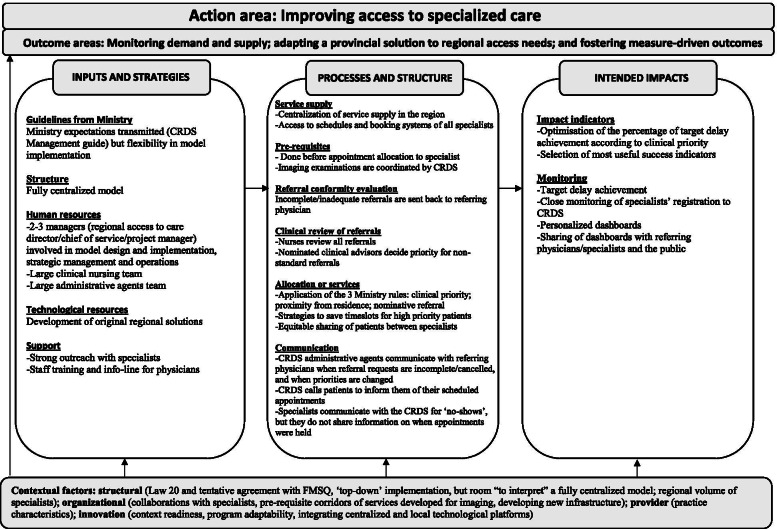
Region B (fully centralized model)

**Fig. 4:**
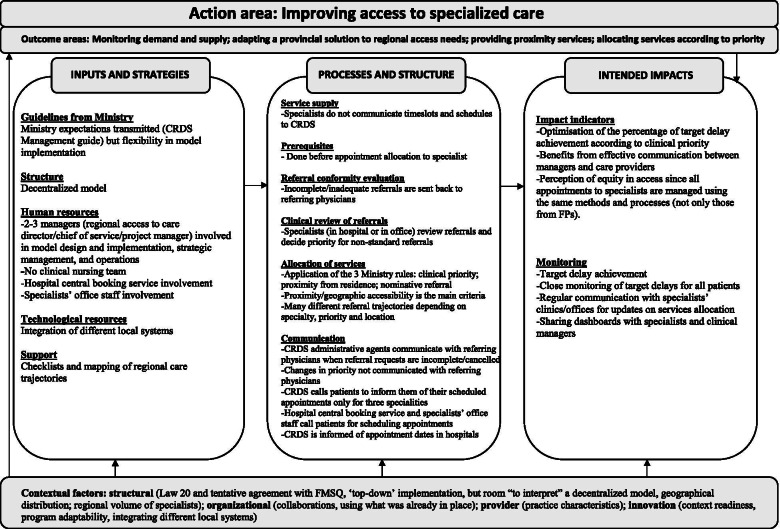
Region C (decentralized model)

Draft logic models were first developed in French (i.e., the first and last authors translated the English codes, sub-themes, and themes), and this so that they may be shared and validated in knowledge dissemination sessions in April 2021, which were attended by four participants representing the three regions (member-checking, [49]). This session also allowed for participants to share what they perceived to be contextual factors influencing the regional variability of the APSS-CRDS models. These contextual factors shared in French were then compared with the English codes by the first and last authors. The contextual factors shared during the dissemination session that matched the ones that were coded were included in the final regional logic models (Figs. 2, 3 and 4) and highlighted in the paper. For the purposes of this article, the validated French logic models were translated into English by the first and the last authors.

## Results

### Description of participants

Nine health administrators were included in the study, 8 of which were females, and 1 male. Their work profiles highlighted distinct functions of the APSS-CRDS. Included in our sample were: a health planner responsible for the regional hospital central booking service (*n* = 1), an administrative technician working to ensure conformity of requests sent to this central booking service (*n* = 1), CRDS project managers (*n* = 2), chief of services at the CRDS (*n* = 3), a head clinical nurse (*n* = 1), and a regional service access planner (*n* = 1). These perspectives allowed us to consider the different regional/local model structures. Participants had more than 2 years of experience with the program, as shared during the interviews, except 2 participants who were new in their role. They were interviewed with a more experienced colleague. Five participants have been involved with the APSS-CRDS program since the program’s implementation in 2016.

### Description of the APSS-CRDS logic models

Figures 2, 3 and 4 highlight our developed and validated logic models for the APSS-CRDS program in three Quebec regions. The headings of each section below represent the themes and sub-themes used for coding and building of the logic models (Figs. 2 to 4). The themes are based on Mitchell and Lewis’ logic model factors (action areas, outcome areas, input and strategies, processes and structures, intended impacts) [41] (Fig. 1; Table 2). Sub-headings are included under each of these themes in the logic models, sub-themes also used for coding and included in the logic models (Figs. 2 to 4). These sub-themes list the components of the APSS-CRDS program [15] and the categories included in Mitchell and Lewis’ logic model [41].

Table 3 includes a summary of the regional logic model differences across each theme and sub-theme (where applicable).

**Table 3:** Differences in the developed APSS-CRDS logic models

Components	Regions		
	**Region A (mostly centralized)**^**a**^	**Region B (fully centralized)**^**a**^	**Region C (decentralized)**^**a**^
**Outcome areas**	Better understanding and clearing already-available wait lists.	Fostering measure-driven outcomes.	Providing proximity health services to patients.
**Inputs and strategies**			
*Human resources*	A person responsible for each University Health and Social Services Center (French: *Centre intégré universitaire de santé et de services sociaux*, CIUSSS).		Staff from the local hospitals’ scheduling centers and the offices of doctors working in the community.
	Clinical nursing team.	Clinical nursing team.	
	Specialist medical advisor directly involved in managing requests is appointed by the Quebec Ministry of Health and Social Services.	Specialist medical advisor directly involved in managing requests is appointed by the Quebec Ministry of Health and Social Services.	Specialist medical advisors are not appointed by the Quebec Ministry of Health and Social Services but were the regional hospital medical chiefs of staff from different specialists.
*Financial resources*	Financing a pilot project to support clearing wait lists.	Developing one regional technological tool to access and monitor data.	–
*Support*	Question and answer document that was shared online.	Presentations about the APSS-CRDS program to specialists and conducted by managers.	Memory aid on the APSS-CRDS functioning for physicians, which included the region’s health trajectories.
		Trainings on using the APSS-CRDS program were offered to physicians and provided by managers.	
		Informal support to physicians offered by managers (i.e., physicians could contact them through a direct phone line).	
**Processes and structures**			
*Registration (i.e, service supply)*	Supply is mostly centralized: the CRDS has limited access to specialists’ schedules via the regional digital platform, as this set-up is available solely in hospital settings.	Supply is centralized: the CRDS receives specialists’ availability 7 days in advance to booking and has access to the schedules of specialists registered with the CRDS via digital platforms.	Specialists do not share their availability with the CRDS, it will transfer requests for consultations directly to specialists’ practices for appointment scheduling.
			Exception: the CRDS has access to specialists’ schedules in Region C if they practice in hospital settings.
*Pre-requisites*	–	Corridors of services allow for the CRDS to schedule patients’ imaging appointments.	–
*Clinical review of referrals*	Evaluations of referrals conducted by nurses and the specialist medical advisor, who will confirm/decide the clinical priority.	Evaluations of referrals conducted by nurses and the specialist medical advisor, who will confirm/decide the clinical priority.	Evaluations of referrals are conducted by the specialists overseeing the speciality at the hospital or clinic.
*Allocation of services*	‘Niche,’ specialized practices, limiting the ability to equitably assign patients via the CRDS to registered specialists and complicates the assignation of patients with “general” problems.	Focusing on high priority patients (priority B), hence the CRDS reserves specialists’ time slots specifically for these high priority patients.	Focusing on offering services as close to the patient’s place of residence, hence the development of many different referral trajectories depending on the speciality, priority and the service location.
		Ensuring the equitable distribution of requests to registered specialists given that the CRDS has access to all registered specialists’ time slots.	
*Communication*	Communication with referring physicians Changes in priorities are communicated to the referring physicians.	Communication with referring physicians Changes in priorities are communicated to the referring physicians.	–
	Communication with patients The CRDS calls patients to inform them of their scheduled appointment with the specialist.	Communication with patients The CRDS calls patients to inform them of their scheduled appointment with the specialist.	Communication with patients Clinic and hospital scheduling staff majorly call patients to fix specialists’ appointments. The exception is solely for three specialities, where the CRDS calls patients to inform them of their scheduled appointment with specialists.
	The CRDS will call patients on waiting lists to inquire if consultations are still needed.		
	Communication with specialists Specialists communicate with the CRDS for ‘no-show’ patients.	Communication with specialists Specialists communicate with the CRDS for ‘no-show’ patients.	Communication with specialists Information on held appointments is communicated from specialists registered with the CRDS.
	Specialists do not share information with the CRDS on when patient appointments are held.	Specialists do not share information with the CRDS on when patient appointments are held.	
**Intended impacts**			
*Performance indictors*	Eliminating duplication.	Choosing the most useful indicators to ensure reaching outcomes.	Monitoring for completed appointments.
	Better understanding population needs (clinical and cultural) to adapt services and improve understanding of referring physicians’ training needs.		Developing a common language to communicate with physicians about requests meeting target delays.
*Monitoring*		Developed personalized dashboards with aggregated data on demand and target delay achievement.	
	Uses CRDS data to monitor regional specialist staffing plans to inform the Quebec Ministry of Health and Social Services.	Monitors specialists’ CRDS registration.	
	CRDS data shared with administrative staff to increase motivation (i.e., areas of improvement, successes)	Shares dashboards with physicians.	Regularly communicates information on service allocation, but this with specialists’ clinics and offices.

^a^The centralized APSS-CRDS was viewed as the creation of a dedicated structure for processing all referral requests across the respective region, and the decentralized model was implemented to build on existing regional structures (i.e., local hospital scheduling centers)

#### Action and outcome areas

Improving access to specialized health care was the most reported and agreed upon action area, also listed in the CRDS Management Guide [15]. Commonly listed outcome areas included developing and implementing a provincial solution to address regional access needs through one (or a few) centralized access points for processing referrals, as well as monitoring demand and supply, also confirmed by the CRDS Management Guide [15]: “The Ministry [what] they wanted in the first place, I think, was to have data on what goes on between the first and the second line [of services]” (Region B-Participant 1). Differences in outcome areas included Region A’s emphasis on better understanding and clearing already-available wait lists; Region B’s focus on fostering measure-driven outcomes; and Region C’s stress on providing proximity health services to patients.

#### Inputs and strategies

##### Ministry guidelines and structure

Participants across regions highlighted the Quebec Ministry’s leniency in the regions’ ability to develop tailored APSS-CRDS models, albeit respecting certain ministerial guidelines listed in the CRDS Management Guide (i.e., ensuring certain human resources be available for daily operations like verifying referral conformity, validating clinical priorities, monitoring delays) [15]. When discussing the APSS-CRDS program structure, Region A described a mostly centralized model, Region B, a fully centralized model, and Region C, a decentralized model. The centralized APSS-CRDS was viewed as the creation of “a centralized structure with a single number to receive [requests]” (Region B-Participant 1) across the respective region, and the decentralized model was implemented to build on existing regional structures (i.e., regional hospital central booking service), in other words, “to invest the least amount of money in a structure that already exists” (Region C- Participant 1).

##### Technological resources

In all regions, the APSS-CRDS budget allocated by the Quebec Ministry was dedicated primarily to program operation and hiring. In Region A (mostly centralized), funding was also used to fund a pilot project to support the clearing of pre-existing waiting lists (updating lists and checking if services were still needed), which required “[establishments] to call back all the patients for […] whom there was a consultation request that was more than a year old, [to] call those patients to see if the patient still needed an appointment” (Region A-Participant 1). Region B emphasized one regionally developed technological tool to access and monitor data.

##### Human resources

The regional APSS-CRDS models are supported by different regional governance structures, managers, and stakeholders. In general, participants across regions reported a couple of managers (regional access to care director, chief of service, project manager), a specialist medical advisor for each of the 26 specialties, and a team of administrative agents. Other staff members included: 1) staff from local hospital scheduling centers and the offices of specialists working within the community (Region C, decentralized model); and 2) a person responsible in each of Region A’s health organizations, which regroup more health services than do Regions B and C. Regions A and B reported a clinical nursing team. Managers developed and adapted CRDS tools, promoted medical specialists’ registration to the APSS-CRDS program, developed inter-organisational communication means to encourage and support FP participation, as well as monitored the regional program. The project manager worked to develop and implement the regional model and engaged in daily operations: “Our mandate was to build the CRDS and […] to do that, we had to develop a regional governance, but we had to establish links with all our partners in the institutions” (Region A-Participant 2). The specialist medical advisor and administrative staff were directly involved in managing requests: the specialist medical advisor, appointed by each specialty’s medical association in Regions A and B, validated requested changes in the referral priorities and non-standard reasons for referral, and the administrative agents called patients to schedule specialist appointments. In Region C, specialist medical advisors were the regional hospital medical chiefs of staff from the different specialties, and this to preserve “a strong alignment with the medical governance” (Region C- Participant 1).

##### Support

Support provided to referring physicians during implementation differed across regions. Region A prepared a question and answer document that was shared online. The managers in Region B provided training on the CRDS’s use as well as informal support (i.e., direct telephone line). Region C developed a memory aid on CRDS functioning, which included the region’s health trajectories.

#### Processes and structure

##### Pre-requisites, referral conformity evaluation, and clinical review of referrals

FPs fill out and submit requests for initial consultations with specialists using a standardized form with predefined prioritization levels according to listed reasons for consultations. Clinical priorities with target wait time are: 1) urgent cases: patients should be directed to emergency; 2) priority A patients: to be directed to the on-call local hospital specialist via a direct telephone call; 3) priority B patients: to be seen in < 10 days; 4) priority C patients: to be seen in < 28 days; priority D patients: to be seen in < 3 months; and 5) priority E patients: to be seen in < 12 months [15]. Forms are sent to the CRDS by regionally developed technological platforms or by fax, where they are reviewed for conformity in all three regions by administrative agents and then sent back to the referring physician for missing information. Evaluations for clinical conformity (reasons for referral and priority levels) are conducted in Regions A and B by nurses in each specialty, and the specialist medical advisor will confirm/decide the clinical priority for non-standard referrals (i.e., requested change in priorities and reasons tagged “other”). In Region C, referral reviews are directly conducted by the specialists overseeing the speciality at the hospital or office. Forms also include some pre-requisite tests that patients must undergo prior to receiving an appointment with a specialist, and these are verified by administrative agents in all regions. In Region B, the CRDS developed corridors of services to help fast-track patient pre-requisites: “What we did was that we told the family doctors, when you send us your request to see the specialist, send us the imaging request with it and we will take care of that in imagery” (Region B-Participant 1). In contrast, the CRDS is currently not involved in any pre-requisite booking in Regions A and C.

##### Service supply

Specialists are prompted to register to the CRDS and to provide availability to be “booked” to see patients from the CRDS [15]. The ways in which specialists share their availability with the CRDS differs. In Regions A and B, offer is centralized according to regional particularities. Region B’s service supply is centralized and the CRDS receives specialists’ availability 7 days in advance to booking. Region B has access to the schedules of specialists registered with the CRDS via digital platforms. In Region A, the CRDS has limited access to specialists’ schedules via the regional digital platform and is required to contact individual hospital services/specialist clinics to check availability: “Well in fact, to have the service offer of the current establishments by week […] we take out the available slots for the week [and] I’m talking about one establishment and we have 10 like that […] we will enter in their computer system, after that we enter the appointment in our computer system, we send [the establishment] a fax to confirm the patient’s request [and] if the patient cancels, well we start this process again” (Region A-Participant 2). In Region C, specialists do not share their availability with the CRDS; however, the CRDS has access to full specialists’ schedules in Region C if they practice in hospital settings and will transfer requests for consultation directly to specialists’ offices in the community for appointment scheduling.

##### Allocation to services

Allocation to specialists is assigned based on three rules devised by the Quebec Ministry [15]. Patient assignment to specialists is based on patients’ clinical priorities, place of residence, and/or requests for a specific specialist (i.e., nominative reference). While these rules are followed in the three regions, Region B focuses on patient requests prioritized as B, and the CRDS reserves specialists’ time slots specifically for these high priority patients. Given the centralized nature of Region B’s CRDS, it can ensure an equitable distribution of requests to registered specialists: “In the ministerial standards, they asked us at the CRDS to distribute [requests] equitably between the doctors, but I can tell you that to date things are going well because those who have a lot of availability send [their availability] to us and we try to fill them all” (Region B-Participant 2). The decentralized nature of Region C’s CRDS model focuses on offering services as close to the patient’s place of residence, which translates into the development of many referral trajectories depending on the specialty priority and service location. In Region A, equitably assigning patients via the CRDS to registered specialists proved difficult given the mention of “niche,” specialized practices. Nominative references are allowed by the APSS-CRDS. This may complicate and, in some cases, delay service allocation since the CRDS may not be aware of the nominated specialist schedule, which is monitored only in Region B by “a [regionally] developed tool ‘on the side’” (Region B-Participant 1). If the appointment cannot be offered within the target wait time for the given priority, the referring FP may be offered a time slot with the first available specialist.

##### Communication

In all regions, CRDS administrative agents communicated with referring FPs when requests were incomplete/cancelled. In contrast with Region C, Regions A and B communicate with referring FPs decisions about requested changes in priorities: “The medical adviser analyzes the request, and after that, the specialty nurse will analyze it and we return it to the referring doctor to advise him if he checked C that the medical adviser put it in D […]” (Region B-Participant 2). In both Regions A and B, the CRDS calls the patients to inform them of their scheduled appointment with the specialist. In contrast, hospital scheduling centers and community specialists’ offices call the patients in Region C, except for three specialties where appointments are managed by the CRDS. In Region A, the CRDS calls patients on wait lists to inquire if consultations are still needed. Specialists in Regions A and B communicate with the CRDS for “no show” patients, but do not share information on when appointments were held. In Region C, the CRDS is informed in real time by the computerized hospital scheduling service when appointments are held in hospital settings, and by monthly direct contact with agents in the community offices.

#### Intended impacts

##### Impact indicators

As the main objective of the APSS-CRDS program in all three regions is to facilitate access to specialized health care, their focus was on optimizing the percentage of requests that met target delays according to clinical priority. Regions also had specific focus on certain intended impacts that were not mentioned in the ministerial directives. With Region A’s outcome area focused on clearing waiting lists, intended impacts included eliminating duplication. In addition, this region’s intended impacts included better understanding population needs (clinical and cultural) to adapt services and to improve understanding of referring FPs’ training needs. For example, an added value of being partially centralized is obtaining information on specialist service demand and offer “because we [the CRDS] have the faculties of medicine that write [to us] and say “we would need statistics because we want to know what we should train our doctors on”” (Region A-Participant 1). Region B’s outcome area of fostering measure-driven outcomes translated into an intended impact that focused on choosing the most useful indicators to ensure reaching anticipated effects. Region C’s intended impacts additionally included monitoring for completed appointments and developing a common language to communicate with physicians about requests meeting target delays.

##### Monitoring

All regions reported monitoring for target delay achievement to meet the overall program action area of improving access to specialized health care. Monitoring per region also had certain particularities. The CRDS in Region B monitored specialists’ CRDS registration to adapt the promotion activities. It also developed personalized dashboards with aggregated data on demand and target delay achievement, and these were largely shared with physicians. Similarly, Region C also regularly communicated information on service allocation, but this with specialists’ hospital clinics and offices: “this is where we can discuss with the medical specialists: How can we increase the service offer or how can we reduce the number of incoming requests or what can we do to better plan appointments” (Region C-Participant 1). In Region A, CRDS data was shared with administrative staff to increase motivation (i.e., areas of improvement, successes), and used to monitor regional specialist staffing plans to inform the Quebec Ministry.

#### Contextual factors

##### Structural-level factors

The APSS-CRDS program was implemented in the context of Bill 20 [12, 13] and was therefore implemented from the ‘top-down:’ “Bill 20, the way it was made, is that […] if you don’t take CRDS patients, it will mean that you are not performing well and then if you are not performing, we will cut your salary [and] that forced the specialists to say well I am going to join the CRDS” (Region B-Participant 1). The tentative agreement between the Quebec Ministry and the health specialists’ union encouraged some adjustments to the monitored indicators’ definitions and the timeline in meeting access targets [14]. With this approach, ministerial expectations for the program were transmitted to managers through the CRDS Management Guide [15], which, according to participants, came late considering that regions were required to have functioning APSS-CRDS models when it was launched. Yet, managers interviewed mentioned that they were also left to their own discretion “to interpret” (Region B-Participant 1) certain program components, like managing and processing referral requests through regionally developed infrastructure. This flexibility may help explain 1) the emergence of fully centralized (Region B), mostly centralized (Region A), and decentralized (Region C) models, perhaps developed in accordance with regional vision of mobilizing services and infrastructure already in place and/or developing new infrastructure, and 2) why other outcome areas and intended impacts than those stated in the CRDS Management Guide and included in the Quebec Ministry and the health specialists’ union agreement were listed by regions.

Another structural factor mentioned by interviewees in all regions includes limits to the regional service offer for given specialties, which can influence delays in patient allocation and thus the performance of the CRDS. To account for specialist shortages, Regions A and B’s CRDS can send requests to other regions for a given specialty. However, according to Region A’s interviewees, this may cause service allocation problems in these other regions.

##### Innovation-level factors

Besides the need to develop some sort of centralized access point, participants shared that the Quebec Ministry of Health and Social Services ‘loosely’ guided the development of regional technological platforms for the CRDS to receive and transmit referrals via these access points. This “loose” guideline left room for regional interpretation, which may have allowed for regions to develop technological platforms that accommodated for level of model centralization: “Because I believe in it, the single access point […] but at some point, I think that each of the regions can implement its model” (Region C-Participant 1). Regions A and C integrated a centralized solution with local systems; and Region B developed an original centralized solution. Technological irritants, however, were shared across regions, including limits to the deployed CRDS platform’s connectivity and interoperability with regionally-used technological solutions by physicians. These irritants may influence the program’s ability to monitor the time patients spend on wait lists and the sharing of information, to name a few. Specifically, the CRDS is not able to monitor “in real time” when specialist appointments are held, which can impede on the ministerial action and outcome areas of the APSS-CRDS program in those areas.

##### Organizational-level factors

Regional inter-organizational collaborations, facilitated by the local climate and culture, as well as managers’ leadership styles, emerged as central in implementing the APSS-CRDS program. Participants from Region B shared that a centralized vision of CRDS implementation required linkages between the program’s managers, regional health planners, and physicians, facilitated by the former’s presence at regional meetings to promote the APSS-CRDS and explain its operation. These privileged linkages facilitated physician registration to the CRDS and the centralization of time slots. The CRDS of Regions A and C encouraged pre-existing collaborations and ‘ways of doing’ using existing human resources and infrastructure. In the decentralized model (Region C), it was perceived as a facilitating factor to nominate local hospital medical chief of staff in the different specialties as medical advisors, and this to acknowledge pre-existing regional medical governance. In Region C, the agents of the hospital scheduling system who give appointments manage all specialist demand, and this so as “not clash with the responding physicians” (Region C-Participant 1). In addition to the referrals sent by FPs through the CRDS, they book specialist appointments for referrals from other specialists, emergency departments, as well as follow-up appointments with specialists. This process gives administrative agents in Region C a complete view of the demand and allows them to assign appointments with some level of equity, which defers from other regions where the CRDS only manages referrals from FPs who transit requests through the CRDS. The corridors of services developed in Region B to facilitate patients’ pre-requisite imaging appointments also attest to the value of CRDS managers’ collaborations. This process may help in achieving target delays specifically for priority B patients by ensuring that imaging tests are completed within a given time frame, especially since wait times for pre-requisites required by the CRDS do not always align target wait time according to clinical priority.

##### Provider-level factors

Physician practice characteristics were seen with some specialists’ “niche” practices, particularly emphasized in Region A: “They can afford to say, “Okay, I specialize in gastroenterology, but I just have hepatitis; I don’t see everything” (Region A-Participant 2). While some specialists would register with the CRDS, they would only treat certain conditions, and some might refuse patients if the ailment was too “general.” This practice may be influenced by the strong university hospital center presence in Region A, fostering many ultra-specialized practices.

### Interpretation

The aims of this article were to 1) describe the APSS-CRDS program in three Quebec regions using logic models; 2) compare similarities and differences in the components and processes of the APSS-CRDS models; and 3) explore contextual factors influencing the models’ similarities and differences. We found that the program’s components as recommended by the Quebec Ministry of Health and Social Services in the CRDS Management Guide [15] were included in the three models, but some were interpreted considering regional particularities and flexibility in the guidelines. These components included in the initial program and interpreted depending on regional particularities are patient and specialist registration, management of referral requests, service allocation, and assessment of delays. Contextual factors influencing the components of the models include ministerial and medical associations’ involvement, the context’s readiness for implementation, collaborations, physicians’ practice characteristics, and the program’s adaptability.

This study builds on the interest of reflecting on the implementation of demand and supply management innovations. Many local initiatives were proposed to address local access problems, but there are gaps in the literature around guiding principles on how these innovations could best be implemented [18]. The literature also shows considerable variation in the program effects [50], a knowledge gap that may be addressed by systematic evaluation. In addition, the studied program (the APSS-CRDS) includes and combines many different features of demand and supply management innovations. This study therefore contributes to the understanding of how such a program is organized, integrates into existing structures, activates centralized waiting list mechanisms, and adapts to the context.

The relevance of using logic modeling as a methodological tool in this paper comes from its ability to illustrate the pathways of the demand and supply management program operations [18]. It is thus a useful tool to identify components the intervention includes by obtaining consensus from stakeholders on complex multi-site, multi-actor, multi-impact program functioning, and it supports managers in articulating a vision about how the program will address specific population and system needs. The logic model tool may also help in better understanding the mechanisms to produce effects and the influence of contextual factors on outcomes, as it prepares future evaluation studies for the identification of the program’s core components and on evidence of their effectiveness from a system’s perspective.

The APSS-CRDS program was implemented as a ‘top-down’ approach following a tentative agreement between the Quebec Ministry and the specialists’ union, in contrast to other centralized waiting lists with homogeneous waiting groups, where their suggestion stemmed from healthcare organizations’ needs [19, 34]. In Quebec, the regional allocation of patients via centralized wait lists, statistics on service demand, and healthcare system performance (i.e., meeting target delays for specialists’ consultations) were of government and medical unions’ interest. The APSS-CRDS program was accompanied by physician sanctions when targets were not met under Bill 20, which contradicts literature on providing participation incentives as a facilitating factor in implementing waiting management strategies [35, 36]. Missing from this ‘top-down’ approach in Quebec are consultations with local/regional healthcare and managerial actors, as well as physicians and patients, elements that could influence program implementation, adherence, and utilization. Among the key elements to guide the implementation and management of single-entry models, Lopatina and colleagues [34] identified the importance of diagnosing the problem, identifying a tailored model structure “to address the context of the system and user needs,” and involving “the relevant stakeholders in the design process” (ref, p.968). CRDS managers in all regions mentioned that their roles included “selling” the CRDS to multiple regional actors, including specialists.

Despite the APSS-CRDS program’s initial implementation in the context of Bill 20 in which specialists were subject to wait time targets, specialist participation is currently optional. Complexity in referral pathways through, in the APSS-CRDS’s case, the absence of a single centralized referral model can encourage regional and local particularities in how to refer (ex.: unique referral processes, availability of specialist time slots), where and to whom (ex.: “niche” practices), as well as multiple waiting lists that could result in referral duplicates. Hence, as seen in our models (Regions A and C), “clinicians of the same specialty, working in the same region, may have different approaches to managing referral and wait lists, potentially leading to inequitable and suboptimal patient outcomes” ([41], p.E413). These complexities and particularities are currently common in outpatient clinics [51] and should be considered when assessing the APSS-CRDS’s effects.

However, differences in the referral complexity as well as regional and local referral patterns [51] may increase sense of personal ownership over referrals, and this from the perspective of physicians [52]. These unique regional particularities including availability of human resources and infrastructure may have encouraged the choice of centralized versus decentralized models for the APSS-CRDS, and an outcome area focused on proximity services (Region C). Decentralized service offer may promote proximity health services, which has patient benefits, including encouraged use of services, satisfaction with the health system, and improved health outcomes [53, 54]. A certain level of decentralization may also help to explain why Region A had an intended impact of better understanding population needs to adapt services and physician training, which can foster patient-centered services.

This paper highlights the importance of context readiness for the implementation of programs like the APSS-CRDS. Two contextual factors emerged that challenged the implementation and functioning of the CRDS: the regional service offer in specialties and an appropriate regional technological platform to monitor demand and supply, harmonized with pre-existing tools. Accessing timely specialist care could be challenged by limited specialty offer [4], and without improvements at this structural level, the CRDS may not meet its action area of improved specialized health care specifically for urgent patients. Some regions use their CRDS data to help the Ministry in better understanding their regional demand for certain specialties. Specialty offer may also be limited by the level of participation (e.g., number of registered specialists and number of time slots dedicated to APSS-CRDS patients). Removing some of the CRDS responsibilities with regards to obtaining specialists’ time slots may be warranted. It could be helpful for all specialists to allocate reserved time slots for patient appointments based on regional demand calculations, shown to reduce wait time for services [55, 56]. This strategy may even serve to further adapt supply to the regional demand, which is a continual struggle for the CRDS. Technological solutions to support the CRDS in its operation are also important. The breadth of the responsibility was and continues to be on the CRDS to find and develop solutions that will not only meet ministerial demands, but also respect regional particularities and “match” pre-existing tools. Technological challenges as experienced by the CRDS may compromise the communication between the CRDS and physicians, which can be perceived as a threat to high-quality care [4].

### Future directions

This paper did not explore the APSS-CRDS program’s effectiveness and how contextual variations may influence its intended impacts, nor did it explore how and why referring FPs used the APSS-CRDS program, next steps for the team’s research. With the COVID-19 pandemic and its impact on healthcare staff relocation to COVID-19 tasks and interruption of certain health services [57], it will be important to assess the APSS-CRDS program’s adaptations in that context, and how they influenced input and strategies, processes and structures, and intended impacts [58]. For example, Quebec ministerial recommendations during the peak of the pandemic waves were to prioritize only urgent patients [59]. Given Region B’s centralized supply and strategies for ensuring that time slots are available for priority B patients, it would be valuable to compare its CRDS activities and target delay achievements with other regions that are more decentralized [51]. In addition, it will be important to assess supply and demand for specialized health services during the pandemic context, and this retrospectively, to glean lessons learned for Quebec, other Canadian provinces, and countries in developing strategies to ensure efficient use of limited health services.

### Limitations

We interviewed nine health administrators from three Quebec regions. While we do acknowledge the limited sample size per region, few regional stakeholders were involved in implementing the APSS-CRDS. It was therefore difficult to interview many people who had knowledge about its implementation in the beginning phases. In addition, interviewees per region confirmed particularities of the APSS-CRDS implementation and new information did not emerge during the knowledge dissemination sessions [60]. Our findings also show that we were able to explore diverse types of CRDS structures (fully centralized, mostly centralized, and decentralized) by interviewing nine health administrators, models that may be reflective of practices across Quebec.

## Conclusion

To our knowledge, our study is the first in Quebec to better understand the implemented APSS-CRDS models and their operation, important in the context of long wait times for speciality consultations. Our findings show the APSS-CRDS program’s regional variability, which we explored by discussing regional contextual factors. Findings can inform Quebec and other decision-makers on ways to improve the APSS-CRDS’s design and contextual factors that may impede on or promote the achieving the program’s anticipated effects (e.g., regional demand for speciality consultations, technological tools that are not harmonized, etc.). Our findings may be useful to other Canadian and country decision-makers interested in implementing similar regional and centralized access points for specialized health services via primary care, and this by considering context in both the design and implementation of standardized programs, as contextual factors were shown to influence the interpretation of APSS-CRDS program components included in the initial program design.

## Supplementary Information

**Additional file 1.** Interview Guide – Health Managers.

## Data Availability

The datasets generated and analysed during the current study are not publicly available due to confidentiality reasons (i.e., identification of the study participants and study regions) but are available from the corresponding author on reasonable request.

## Abbreviations

APSS: Accès priorisé aux services spécialisés
CRDS: Centre de répartition de services
FP: Family physician
FPs: Family physicians
CIUSSS: Centres intégrés universitaires de santé et de services sociaux
CISSS: Centres intégrés de santé et de services sociaux
