# Supplementary material for: Exploring the implementation and underlying mechanisms of centralized referral systems to access specialized health services in Quebec
Source: BMC Health Serv Res. 2021 Dec 16;21:1345. doi: 10.1186/s12913-021-07286-3 (PMC8674406; doi:10.1186/s12913-021-07286-3)
Supplement: Supplementary file 1 — Additional file 1. Interview Guide – Health Managers. [file 12913_2021_7286_MOESM1_ESM.pdf]

Action and Outcome Areas

1. What changes do you think the implementation of the APSS-CRDS program in your establishment/region was intended to generate? (Probes: effect on the health system, patients, etc.)

Input and Strategies

2. Can you please describe how the APSS-CRDS program was implemented in your setting?

- What resources (human, financial, material) does the program rely on?
- Who funds the program and how is the funding used?
- What type of support or training have you received to implement the program?
- What are the program's governance structures (local, regional, and provincial)?
- How does the APSS-CRDS program operate in your region (title and role of key stakeholders involved)?
- How was the implementation of the APSS-CRDS program received in your region? (probes: readiness of the context, clinical utility of the intervention)

3. During the APSS-CRDS program's implementation, what were the strategies to promote it (or discourage its use, if any) to family physicians and/or specialists?

4. Were there any strategies to discourage the use of the APSS-CRDS by family physicians and/or specialists?

5. What tools (computer-based or otherwise) were available to you for the implementation of the APSS-CRDS?

Processes and Structures

6. Can you please describe how you receive referral requests?

- How are referrals sent to you?
- How does the APSS-CRDS proceed if the referral requires additional information (administrative or clinical)?
- How are referral requests with a reason tagged 'other' than the pre-established list of clinical conditions handled?
- How are referrals with a level of prioritization listed by the referring physician that is different than the one recommended by CRDS staff handled?
- How are referring physicians supported in using the APSS-CRDS (training, support, decisional aid, etc.)?

7. Can you please describe how CRDS appointments are assigned to patients?

- How does patient prioritization work?
- How do patients and specialists 'match'?

- How is the regional service offer known by the CRDS?
- Are there any criteria for matching patients with specialists, such as patients' place of residence or patient preferences?
- How is the waiting list managed?
- How are patients notified when they have an appointment with a specialist?
- Can patients receive information about their referral status?
- Does the CRDS receive any information about patients once they are 'matched' with specialists (confirmation that the appointment took place, feedback on the appropriateness of the referral, etc.)?
- How is clinical information about the consultation with the specialist communicated with the referring physician?
- How do you proceed when a patient's referral through the CRDS requires prior diagnostic tests or other pre-requisites?
- Who is responsible for managing these diagnostic tests or other prerequisites?
- How does the CRDS follow-up with referring physicians (and how often, or why not)? Do you proceed to follow up with referring physicians?
- Can referring physicians obtain information on the status of their referrals to specialized services?

Context

8. What factors at the provincial or regional level may have influenced the implementation of the APSS-CRDS program? (Probes: factors related to the social, geographic, political, and/or economic contexts, etc.)

9. What factors that are specific to your establishment (CIUSSS/CISSS, etc.) may have influenced the implementation of the APSS-CRDS program? (Probes: leadership, organizational culture, staff attitudes, etc.)

10. What physician characteristics (referring physicians and specialists) may have influenced the implementation of the APSS-CRDS program? (Probes: independence in practice, attitudes towards the intervention, level of confidence in using new practices, etc.)

11. Aside from consideration of their clinical condition, how might patients influence the use of CRDS? (Probe: satisfaction, perceived benefits)

12a. How do you think the way in which the APSS-CRDS program was designed may influence its implementation? (Probes: benefits of using the CRDS over existing practices, quality of evidence using to inform the CRDS, etc.)

Impacts

13. What type of monitoring is conducted by the APSS-CRDS in your region? What type of systematic tracking is available for different indicators - which ones and by whom is this monitoring conducted?

14. How has the APSS-CRDS program impacted access to specialized services since its implementation?

- What improvements could be made to the APSS-CRDS program?
